# Hyper-truncated Asn355- and Asn391-glycans modulate the activity of neutrophil granule myeloperoxidase

**DOI:** 10.1074/jbc.RA120.016342

**Published:** 2020-12-10

**Authors:** Harry C. Tjondro, Julian Ugonotti, Rebeca Kawahara, Sayantani Chatterjee, Ian Loke, Siyun Chen, Fabian Soltermann, Hannes Hinneburg, Benjamin L. Parker, Vignesh Venkatakrishnan, Regis Dieckmann, Oliver C. Grant, Johan Bylund, Alison Rodger, Robert J. Woods, Anna Karlsson-Bengtsson, Weston B. Struwe, Morten Thaysen-Andersen

**Affiliations:** 1 Department of Molecular Sciences, Macquarie University, Sydney, New South Wales, Australia; 2 Biomolecular Discovery Research Centre, Macquarie University, Sydney, New South Wales, Australia; 3 Cordlife Group Limited, Singapore, Singapore; 4 Department of Chemistry, University of Oxford, Oxford, United Kingdom; 5 Department of Physiology, University of Melbourne, Melbourne, Victoria, Australia; 6 Department of Rheumatology and Inflammation Research, Institute of Medicine, Sahlgrenska Academy, University of Gothenburg, Gothenburg, Sweden; 7 Complex Carbohydrate Research Center, University of Georgia, Athens, Georgia, USA; 8 Department of Oral Microbiology and Immunology, Institute of Odontology, Sahlgrenska Academy, University of Gothenburg, Gothenburg, Sweden; 9 Department of Biology and Biological Engineering, Chalmers University of Technology, Gothenburg, Sweden

**Keywords:** myeloperoxidase, *N*-glycosylation, neutrophil, granule, biosynthesis, activity, inhibition, ceruloplasmin, granulopoiesis, degranulation, AUC, area-under-the-curve, Az granule, azurophilic granule, Az-MPO, azurophilic granule-resident MPO, BN, band neutrophil, CD, circular dichroism, CytB/I, cytochalasin B and ionomycin, Dg-MPO, degranulated MPO, EIC, extracted ion chromatogram, Endo H, endoglycosidase H, ER, endoplasmic reticulum, Fuc (F), α-L-fucose, Ge granule, gelatinase granule, Ge-MPO, gelatinase granule-resident MPO, GlcNAc, *N*-acetyl-β-D-glucosamine, H_2_O_2_, hydrogen peroxide, HOCl, hypochlorous acid, KRG buffer, Krebs-Ringer buffer with glucose, LC-MS/MS, liquid chromatography tandem mass spectrometry, LDH, lactate dehydrogenase, LFQ, label-free quantitation, Man, α/β-D-mannose, MD, molecular dynamics, MM, metamyelocyte, MOI, multiplicity-of-infection, MPO, myeloperoxidase, nMPO, neutrophil-derived myeloperoxidase (unfractionated), PDB, Protein Data Bank, PGC, porous graphitised carbon, PM, promyelocyte, PMN, polymorphonuclear cell (neutrophil), PMN-nMPO, myeloperoxidase from derived from resting (circulating) neutrophils, RMSD, root mean squared deviation, RMSF, root mean squared fluctuation, SD, standard deviation, Se/Pl, secretory vesicle and plasma membrane fraction, Se/Pl-MPO, secretory vesicle/plasma membrane-resident MPO, SN, maturing neutrophil with segmented nuclei, Sp granule, specific granule, Sp-MPO, specific granule-resident MPO, TMB, 3,3ʹ,5,5ʹ-tetramethylbenzidine

## Abstract

Myeloperoxidase (MPO) plays essential roles in neutrophil-mediated immunity *via* the generation of reactive oxidation products. Complex carbohydrates decorate MPO at discrete sites, but their functional relevance remains elusive. To this end, we have characterised the structure–biosynthesis–activity relationship of neutrophil MPO (nMPO). Mass spectrometry demonstrated that nMPO carries both characteristic under-processed and hyper-truncated glycans. Occlusion of the Asn355/Asn391-glycosylation sites and the Asn323-/Asn483-glycans, located in the MPO dimerisation zone, was found to affect the local glycan processing, thereby providing a molecular basis of the site-specific nMPO glycosylation. Native mass spectrometry, mass photometry and glycopeptide profiling revealed significant molecular complexity of diprotomeric nMPO arising from heterogeneous glycosylation, oxidation, chlorination and polypeptide truncation variants and a previously unreported low-abundance monoprotomer. Longitudinal profiling of maturing, mature, granule-separated and pathogen-stimulated neutrophils demonstrated that nMPO is dynamically expressed during granulopoiesis, unevenly distributed across granules and degranulated upon activation. We also show that proMPO-to-MPO maturation occurs during early/mid-stage granulopoiesis. While similar global MPO glycosylation was observed across conditions, the conserved Asn355-/Asn391-sites displayed elevated glycan hyper-truncation, which correlated with higher enzyme activities of MPO in distinct granule populations. Enzymatic trimming of the Asn355-/Asn391-glycans recapitulated the activity gain and showed that nMPO carrying hyper-truncated glycans at these positions exhibits increased thermal stability, polypeptide accessibility and ceruloplasmin-mediated inhibition potential relative to native nMPO. Finally, molecular modelling revealed that hyper-truncated Asn355-glycans positioned in the MPO-ceruloplasmin interface are critical for uninterrupted inhibition. Here, through an innovative and comprehensive approach, we report novel functional roles of MPO glycans, providing new insight into neutrophil-mediated immunity.

The myeloid lineage-specific myeloperoxidase (MPO) is an essential component of the innate immune system associated with many pathologies including cardiovascular diseases (1, 2), rheumatoid arthritis (3) and multiple sclerosis (4). Mutations in human *MPO* and genetic ablation in mice have repeatedly been linked to enhanced pathogen infection (5, 6, 7, 8).

While our knowledge of MPO has improved considerably over the past decades (9, 10, 11), new fascinating facets of MPO biology continue to emerge (12, 13, 14). Facilitated by its peroxidase activity, MPO is known to catalyse the formation of reactive oxidation products including hypochlorous acid (HOCl) from chloride ions (Cl^−^) and hydrogen peroxide (H_2_O_2_), substrates found in the maturing phagosomes (15, 16). From the principal residence within the azurophilic (Az) granules, and to a lesser extent within the specific (Sp) and gelatinase (Ge) granules and in secretory vesicles and the plasma membrane (collectively referred to as the Se/Pl fraction) of neutrophils (17, 18), MPO is emptied into phagosomes or secreted through degranulation upon neutrophil activation (19, 20). Within the phagosome, MPO generates highly reactive hypohalous acids and nitrogen dioxide, which readily react to form diverse reactive oxygen species, key microbicidal and immune-regulatory products of the neutrophil MPO-halide system.

The complex biogenesis and maturation of MPO have been intensely studied (12, 21). Briefly, human MPO is translated as a single 80 kDa signal peptide-containing polypeptide chain. This preproMPO form undergoes extensive proteolytic processing initiated by the removal of the signal peptide to form apoproMPO. The enzymatically active proMPO form is rapidly generated *via* heme acquisition in the endoplasmic reticulum (ER) (22). Protease-driven polypeptide processing then removes the propeptide and an internal hexapeptide, which separates the light (α, 12.5 kDa) and heavy (β, 60–65 kDa) chains that remain covalently linked *via* the catalytic heme. His261 and Arg405 (UniProtKB numbering used throughout the paper) are key catalytic residues of the active site that is formed around the heme moiety positioned between the α- and β-chains. By mechanisms that remain unclear, but presumably occurring within the Golgi, the α- and β-chains are further processed and extensively post-translationally modified before and/or after the formation of the mature MPO diprotomer (ααββ, ∼150 kDa) that is connected by a Cys319-Cys319 bridge (12).

The high-resolution crystal structures of neutrophil-derived MPO (nMPO) with and without cognate and artificial ligands have improved our knowledge of the structure of MPO, the position of the heme and other hetero-atoms essential for enzymatic activity (*e.g.*, Ca^2+^) and the interactions to the endogenous inhibitor ceruloplasmin (21, 23, 24, 25, 26, 27). Thirty years ago, Nauseef and colleagues reported on the existence of five sequons for asparagine-linked (*N*-linked) glycosylation (Asn323, Asn355, Asn391, Asn483, Asn729) all located within the mature MPO β-chain (28, 29). Most MPO crystal structures harbour remnants of *N*-glycan moieties, but our structural knowledge of the MPO glycosylation remains immature since complex carbohydrates are usually incompletely resolved with X-ray crystallography.

Four studies have reported on nMPO glycosylation over the past decade (30, 31, 32, 33). In those studies, mass-spectrometry-based glycopeptide analyses detailed the site-specific monosaccharide compositions decorating nMPO. Compositions corresponding to uncommon chitobiose core- and paucimannosidic-type *N*-glycans as well as more conventional oligomannosidic- and complex-type *N*-glycans were reported, but neither the glycan fine structures and occupancy at each site nor the biosynthesis and functional relevance of the *N*-glycans carried by nMPO distributed across the neutrophil granules were addressed. In fact, the molecular-level knowledge of the roles of nMPO glycosylation is critically missing despite recent reports suggesting that MPO glycans regulate E-selectin binding (34), antigenicity (35, 36) and MPO enzyme activity (31).

Here, we address this fundamental knowledge gap by characterising the structure–biosynthesis–activity relationship of nMPO and by profiling the dynamic expression, protein processing and site-specific glycosylation of MPO from maturing, mature, granule-separated and activated neutrophils. Integration of mass spectrometry, computational and biochemical assays was used to provide new insight, from atomic to macromolecular detail, into the intriguingly complex MPO glycobiology.

## Results and discussion

### Comprehensive characterisation of neutrophil-derived myeloperoxidase

**Figure 1:**
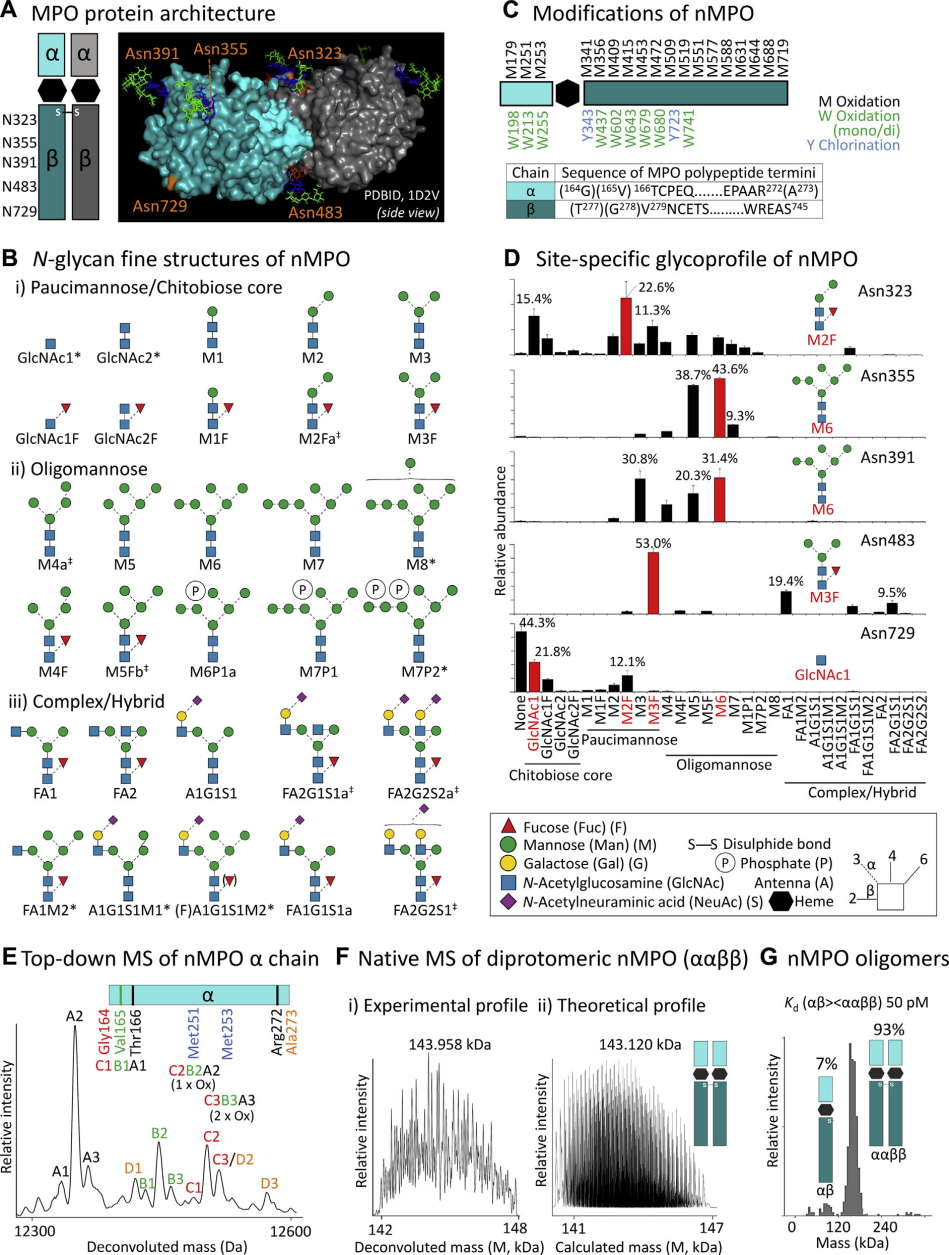
**Comprehensive characterisation of neutrophil-derived myeloperoxidase (nMPO)**. *A*, protein architecture of the heme-containing diprotomeric MPO (ααββ) (left). Each β-chain harbours five *N*-glycan sequons (*orange*) mapped on diprotomeric MPO (PDBID, 1D2V). Asn729 was left unoccupied while characteristic glycans were added *in silico* to other sites (see *D*). *B*, fine structures and short-hand nomenclature of the nMPO *N*-glycans (Fig. S1, Table S1 and Data S1). ∗Several hyper-truncated and/or trace *N*-glycans were only observed at the peptide level. ^‡^Multiple glycan isomers (designated a, b) were identified. See key for glycan symbol nomenclature and linkage representation (37). *C*, non-glycan modifications of nMPO including Met (*black*) and Trp (*green*) mono- and di-oxidation, Tyr (*blue*) mono-chlorination and polypeptide truncation variants (Fig. S2 and Data S2, *A–B*). *D*, site-specific *N*-glycoprofile of nMPO (Fig. S3, Table S2 and Data S3, *A–E*). Prominent *N*-glycans at each site are in red. Data plotted as mean ± SD, n = 3, technical replicates. *E*, intact mass analysis revealed limited α-chain heterogeneity arising from polypeptide truncation and Met251 and Met253 oxidation variants as supported by peptide data (Table S3*A*). *F*, *i*–*ii*, native MS of whole unfractionated nMPO revealed significant complexity of diprotomeric nMPO (ααββ) complexes that matched the expected profile generated from approximately 300,000 predicted glycoproteoforms (Table S3*B*). *G*, mass photometry revealed the existence of a previously unreported low-abundance monoprotomer (7%) and a high-abundance diprotomer (93%) of nMPO.

We first sought to unravel the molecular complexity of the heme-containing MPO (ααββ), which adopts a complex diprotomeric structure comprising two identical αβ protomers, Figure 1*A*. Each β-chain harbours five sequons (Asn323, Asn355, Asn391, Asn483, Asn729) enabling extensive *N*-glycosylation of the protein surface. We applied our established PGC-LC-MS/MS glycomics platform to fully define the molecular structure of all *N*-glycans present on nMPO, including identification of isomeric glycans, Figure 1*B*. Documentation for all reported structures has been provided, Figure S1, Table S1 and Data S1. Uncommon monoantennary complex-type (FA1G1S1a), under-processed oligomannosidic (M5–M6) and hyper-truncated paucimannosidic (M2Fa-M3F) and chitobiose core-type (GlcNAc1–GlcNAc1F) structures were found to be characteristic *N*-glycans of nMPO. These *N*-glycans are congruent with structures residing in the neutrophil granules (38) and those carried by other granule-resident glycoproteins including neutrophil cathepsin G and elastase (32, 39, 40). No MPO *O*-glycans were detected in the glycomics datasets.

Many non-glycan nMPO modifications including a total of 18 Met and nine Trp mono-/di-oxidations, two sites for Tyr mono-chlorination and several polypeptide truncation variants of both the α- and β-chains were identified using sensitive peptide profiling facilitating a near-complete sequence coverage of nMPO (α-/β-chain, 100.0%/99.8%), Figure 1*C*, Figure S2 and Data S2, *A*–*B*. The observed polypeptide hyper-oxidation, which is known to be mediated by the reactive oxidising agents produced by MPO (30), plays recognised roles in neutrophil function (41).

Further, site-specific glycoprofiling revealed an extensive micro- and macro-heterogeneity of all five *N*-glycosylation sites of nMPO, Figure 1*D*, Figure S3, Table S2 and Data S3, *A–E*. In agreement with the previous four studies reporting on the site-specific monosaccharide compositions of nMPO (30, 31, 32, 33), we found that Asn323 and Asn483 predominantly carry paucimannosidic-type *N*-glycans (M2F–M3F), Asn355 and Asn391 carry oligomannosidic-type *N*-glycans (M5–M6) while Asn729 is largely unoccupied or carries chitobiose core-type (GlcNAc1–GlcNAc1F) *N*-glycans. We identified low levels of mannose-6-phoshorylation on nMPO relative to a recent study (33). Such discrepancies may arise from differences in the methods used for the purification and/or analysis of the glycoprotein. Notably, we observed very similar glycosylation of all five sites of nMPO arising from different sources including from unperturbed neutrophil extracts (see detailed comparisons below) and acquired using different techniques supporting an unbiased isolation and characterisation of nMPO.

Intact mass analysis revealed limited α-chain heterogeneity (12,350–12,550 Da) arising from relatively few polypeptide truncation and oxidation variants, presumably Met251 and Met253, as supported by peptide data, Figure 1*E* and Table S3*A*. The variable protein modifications identified at the glycopeptide and peptide level were however reflected in our native MS analysis of whole unfractionated nMPO as demonstrated by significant molecular complexity of diprotomeric nMPO (ααββ, 141.5–148.0 kDa, apex 143,958 Da), which matched a published lower-resolution profile of intact nMPO (apex 144,180 Da) (33) as well as a theoretical profile generated from ∼300,000 glycoproteoforms predicted based on quantitative peptide data (140.5–147.0 kDa), Figure 1*F* and Table S3*B*. Native MS also indicated the existence of an nMPO monoprotomer with a slightly lower-than-expected molecular mass (αβ, 70–73 kDa, data not shown). Cooled non-reductive SDS-PAGE followed by peptide profiling supported the presence of a maturely processed low-abundance nMPO monoprotomer, Figure S4. Single-molecule mass photometry, a method capable of quantifying the assembly, binding affinities and kinetics of protein complexes (42, 43, 44) confirmed that nMPO exists as a low-abundance monoprotomer (αβ, 7%) and high-abundance diprotomer (ααββ, 93%) with an apparent mono-/diprotomer (αβ/ααββ) *K*_d_ of ∼50 pM, Figure 1*G*. The biological role of the maturely processed monoprotomeric MPO, which differs from the secreted monoprotomeric proMPO reportedly elevated in blood in cardiovascular disease (45, 46), remains unknown.

### Tertiary and quaternary structural features explain the site-specific *N*-glycosylation of MPO

**Figure 2:**
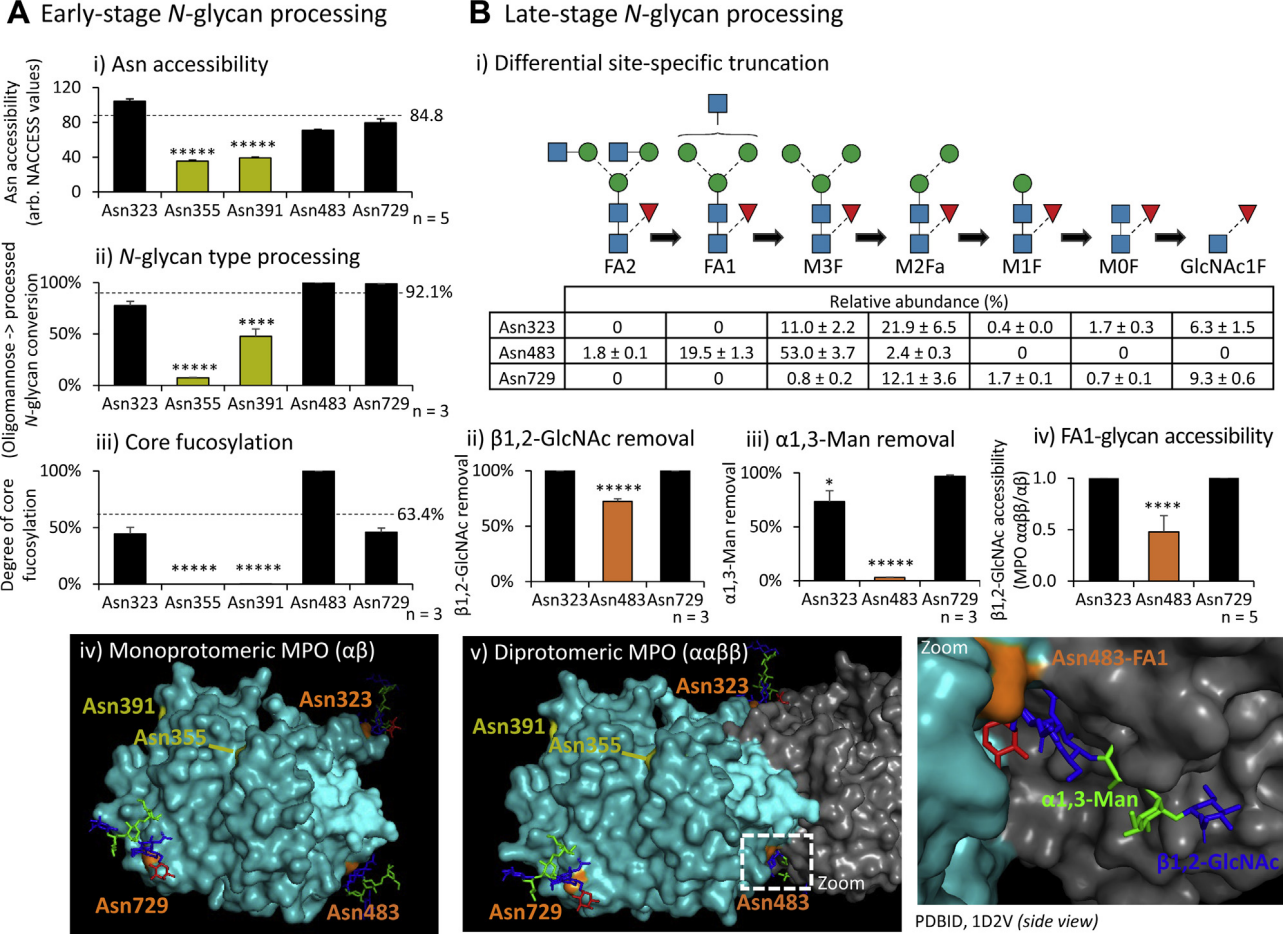
***In silico*–assisted association studies inform on the molecular basis for the site-specific *N*-glycosylation of MPO**. *A*, *in silico*–guided investigation of maturely folded monoprotomeric MPO (as it appears in the early-stage processing) revealed correlations between (*i*) the Asn accessibility (n = 5, different crystal structures) and the degree of (*ii*) *N*-glycan type processing plotted as the oligomannose-to-processed *N*-glycan conversion (n = 3, technical replicates) and (*iii*) core fucosylation determined from possible FUT8 substrates based on nMPO glycopeptide profiling and solvent accessibility data (n = 3, technical replicates) (Fig. 1*D*, Table S2 and Table S4*A*). The occluded Asn355 and Asn391 (*green bars*) carried mostly under-processed oligomannosidic *N*-glycans and afucosylated *N*-glycans. Mean values for the accessible sites (Asn323, Asn483, Asn729) (*broken lines*) were statistically compared with Asn355 and Asn391. *iv*, occluded Asn355 and Asn391 (*green*, depicted without glycans for clarity) and accessible Asn323, Asn729 and Asn483 (*orange*) were mapped on monoprotomeric MPO (PDBID, 1D2V). *B*, *i*, exploration of the late-stage glycan processing of maturely folded MPO involving the *N*-glycan truncation pathway (48, 49) demonstrated less truncation of the Asn483-glycans and partly Asn323-glycans as measured by the lower removal efficiency of terminal residues including (*ii*) β1,2-GlcNAc (n = 3, technical replicates) and (*iii*) α1,3-Man residues relative to Asn729-glycans based on nMPO glycopeptide data (n = 3, technical replicates) and (*iv*) a reduced β1,2-GlcNAc accessibility of FA1-conjugated Asn483 (*orange*) upon MPO dimerisation as determined *in silico* (n = 5, different crystal structures) (Table S4*B*). Statistical comparisons were made to Asn729. *v*, illustration of the dimerisation-dependent occlusion of the Asn483-glycan on diprotomeric MPO (1D2V). Contact between (and hence masking of) the Asn483-FA1 glycan and the protein surface of the other αβ-protomer was observed (see zoom). The Asn323-glycan positioned near the diprotomeric interface was partially affected by MPO dimerisation. For all panels, data plotted as mean ± SD, ∗*p* < 0.05, ∗∗*p* < 0.01, ∗∗∗*p* < 0.005, ∗∗∗∗*p* < 0.001, ∗∗∗∗∗*p* < 0.00005. See Figure 1 for key.

The presence of vastly different glycan structures across glycosylation sites on a given protein is interesting because the protein experiences the same ensemble of glycan-processing enzymes as it traffics the ER-Golgi pathways. We aimed to identify the biochemical basis for this heterogeneity by first investigating using *in silico* approaches the mechanisms impacting the early-stage glycoprotein processing where MPO enters the *cis*-Golgi as a fully folded monoprotomer (12). As we have observed for other mammalian glycoproteins (47), strong associations between the Asn accessibility on the MPO surface and the degree of *N*-glycan type processing and core fucosylation were identified, Figure 2*A*, Table S2 and Table S4*A*. The relatively occluded Asn355- and Asn391-glycosylation sites carried under-processed oligomannosidic-type and afucosylated *N*-glycans as opposed to the surface exposed sites (Asn323, Asn483, Asn729) (*p* = 3.5 × 10^−13^ and 5.7 × 10^−13^, respectively) carrying significantly more processed *N*-glycans (*p* = 4.7 × 10^−4^ and 7.6 × 10^−8^) and core fucosylation (both *p* = 1.3 × 10^−7^).

Further, *in silico*–assisted exploration of the late-stage MPO processing within the *N*-glycan truncation pathway, a glycan-processing pathway highly active in neutrophils (39, 48, 49), demonstrated that the Asn483-glycans (and partly the Asn323-glycans) undergo less truncation relative to the Asn729-glycans, Figure 2*B*i. The lower removal efficiency of the outer β1,2-GlcNAc and α1,3-Man residues of the Asn483-glycans relative to the Asn729-glycans (*p* = 3.5 × 10^−5^ and 1.1 × 10^−8^, respectively) correlated with a reduced solvent accessibility of the β1,2-GlcNAc of Asn483-FA1 glycans upon MPO dimerisation (*p* = 7.6 × 10^−5^), Figure 2*B*ii–iv and Table S4*B*. The dimerisation-dependent occlusion of the Asn483-glycans could be observed by the contact between (and masking of) the Asn483-FA1 glycan and the surface of the other αβ-protomer, Figure 2*B*v. Intuitively, occlusion of the Asn483-glycans restricts the *N*-acetyl-β-hexosaminidase (P06865/P07686) and lysosomal α-mannosidase (O00754), key glycoside hydrolases of the truncation pathway (48, 49), to access their glycan substrates and, in turn, less efficiently catalyse β1,2-GlcNAc and α1,3-Man removal of the Asn483-FA1 glycans. The Asn323-glycan positioned near the MPO diprotomeric interface was partially affected by MPO dimerisation as indicated by a modestly impaired α1,3-Man removal of Asn323-glycans (*p* = 0.014, Asn323 *versus* Asn729). The dimerisation-dependent protection of the Asn323- and Asn483-glycans from hydrolase-mediated truncation was supported by glycopeptide profiling of gel-separated mono- and diprotomeric nMPO, Figure S4*A*. This analysis confirmed that the outer β1,2-GlcNAc of the Asn323- and Asn483-glycans was more efficiently removed of mono- rather than diprotomeric nMPO (*p* = 0.013 and 0.032, respectively), Table S5, *A–B*. Similar protective effects could be observed for the Asn323-glycans of diprotomeric MPO exhibiting a lower α1,3-Man removal efficiency relative to monoprotomeric MPO (*p* = 0.008). Importantly, similar processing of the Asn729-glycans, distal to the dimerisation interface, was observed on mono- and diprotomeric nMPO (*p* ≥ 0.05). Collectively, and in line with current literature (12), our *in silico*–assisted analyses show that MPO dimerisation takes place immediately before or quickly after MPO arrives in the neutrophil granules, a maturation step that affects the processing of the Asn323- and Asn483-glycans positioned at the MPO diprotomeric interface. Follow-up studies aiming to generate direct biochemical evidence of MPO from subcellular-fractionated neutrophils are warranted to support our *in silico*–guided observations of processes relevant to the biosynthesis of MPO and the site-specific processing of its *N*-glycans.

### Dynamics, distribution, processing and glycosylation of MPO across neutrophil life stages

**Figure 3:**
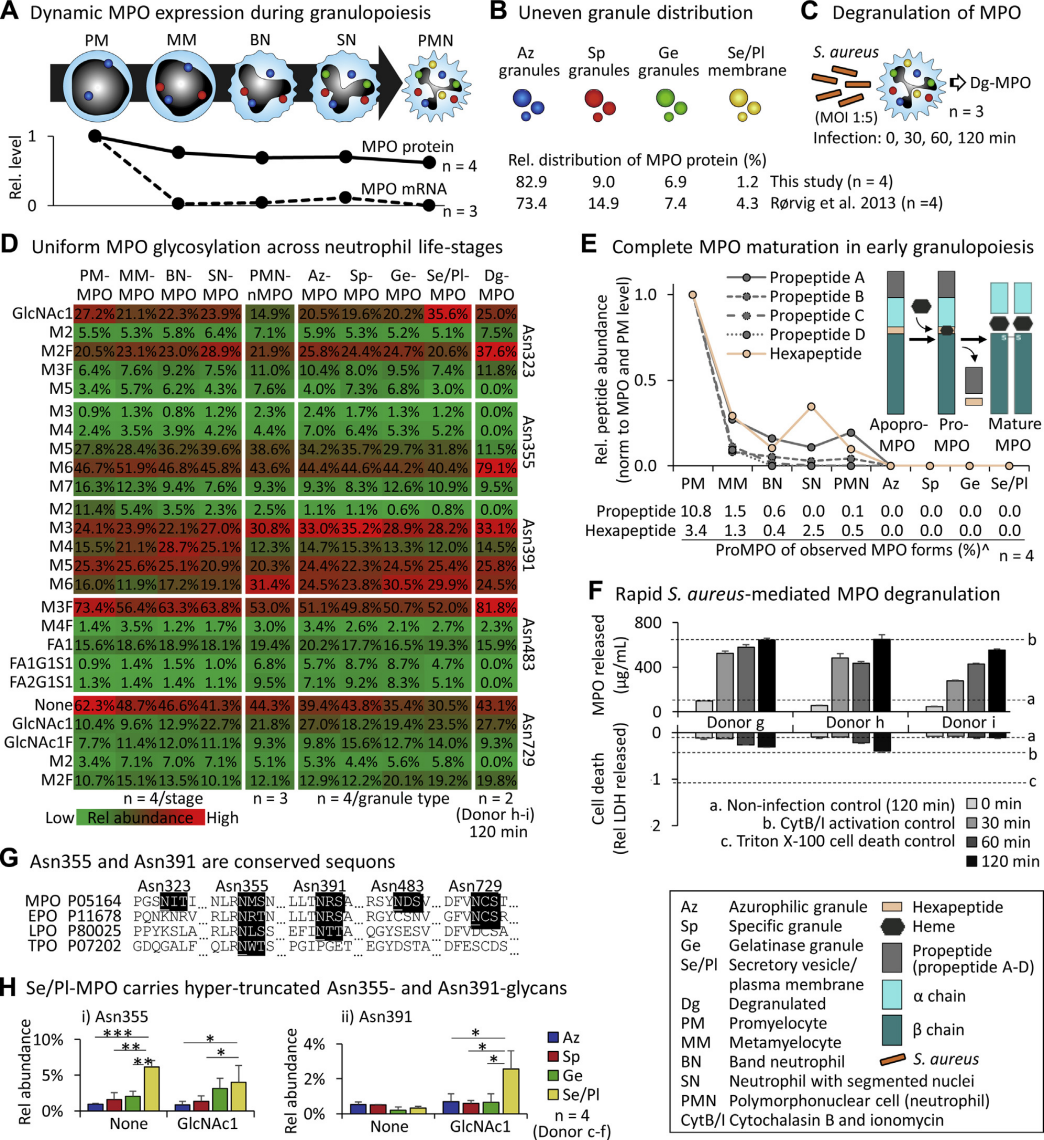
**Dynamics, granule distribution, polypeptide processing, site-specific glycosylation and degranulation of MPO across the neutrophil life stages.***A*, dynamic expression of MPO mRNA (n = 3, biological replicates) and protein (n = 4, biological replicates) during granulopoiesis. The relative MPO expression levels, normalised to the PM-stage, were established by data re-interrogation (50). See key for nomenclature. *B*, quantitative proteomics of granule-separated neutrophils (n = 4, biological replicates) showed an uneven MPO distribution across granules congruent with literature (18). *C*, *S. aureus*-mediated neutrophil activation. Longitudinal profiling of the levels and glycosylation of released Dg-MPO (see panel *F*, *D* for data and controls). *D*, site-specific glycoprofiling of MPO from maturing, resting (PMN-nMPO, see Fig. 1*D*), granule-separated, and activated neutrophils (average of n = 2–4 plotted, number of biological replicates is indicated). Prominent *N*-glycans at each site were plotted (Fig. S5 and Table S6). The MPO glycosylation of maturing neutrophils was determined by data re-interrogation (50). See key for intensity scale and biological replicates and Figure 1 for short-hand nomenclature of glycans. *E*, longitudinal tracking of the proMPO-to-MPO conversion within maturing, mature, and granule-separated neutrophils. Relative levels of peptides arising from the proMPO regions (normalised to PM-stage) were plotted. ˆProMPO levels were determined from the intensity of peptide pairs arising from proMPO and mature MPO. The data do not discriminate apoproMPO and proMPO. See insert for schematics of MPO polypeptide maturation (average of n = 4, biological replicates). *F*, *S. aureus*-mediated activation of neutrophils (MOI 1:5) demonstrating a rapid time-dependent and cell death-independent degranulation of Dg-MPO as measured by ELISA and LDH release assays. Controls (a–c, mean plotted as broken lines) were included for both assays (n = 3, technical replicates). *G*, sequence alignment showed that Asn355 and Asn391 are conserved sequons across the human peroxidases implying functional importance. See Figure S6 for sequence alignment to mammalian MPO. *H*, hyper-truncated GlcNAcβAsn and absent glycosylation were elevated features at (*i*) Asn355 and (*ii*) Asn391 of Se/Pl-MPO relative to MPO from other granules (n = 4, biological replicates). For all panels, data plotted as mean ± SD. ns, not significant (*p* ≥ 0.05), ∗*p* < 0.05, ∗∗*p* < 0.01, ∗∗∗*p* < 0.005, ∗∗∗∗*p* < 0.001, ∗∗∗∗∗*p* < 0.00005. EPO, eosinophil peroxidase; LPO, lactoperoxidase; TPO, thyroid peroxidase.

We next investigated the possible spatiotemporal expression of nMPO and compartment-specific glycosylation during neutrophil formation and activation. Dynamic expression of MPO mRNA and protein during granulopoiesis was demonstrated by re-interrogation of transcript and proteomics data obtained from maturing neutrophils (50), Figure 3*A*. Consistent with a previous protein profiling study of neutrophil granules (18) and with the targeting-by-timing model that describes the timely packaging of proteins in granules during granulopoiesis (51), we show that MPO predominantly resides in azurophilic (Az, 82.9%) granules while the specific (Sp) and gelatinase (Ge) granules and the secretory vesicles/plasma membrane (Se/Pl) contain less MPO, Figure 3*B*. The neutrophil granule distribution of MPO was determined from four biological replicates using a conventional label-free quantitation (LFQ) proteomics approach in which the LFQ intensities of MPO were normalised across the neutrophil granules. Neutrophils are known to release their proteinaceous granule content *via* degranulation upon stimulation (20). To expand on these findings, we monitored the release and glycosylation patterns of degranulated MPO (Dg-MPO) longitudinally upon neutrophil activation mediated by low-level short-term infection by *Staphylococcus aureus*, an opportunistic pathogen present in neutrophil-rich tissues undergoing inflammation including, for example, the upper respiratory tract of individuals living with cystic fibrosis, Figure 3*C*.

Site-specific glycoprofiling of MPO from maturing, mature (resting), granule-separated and pathogen-activated neutrophils showed that MPO carries relatively similar glycosylation across the neutrophil life stages and under the conditions that were analysed, Figure 3*D*. Similar MPO glycosylation patterns of all five sites were observed across all granule types (correlation coefficients, r > 0.95), which, as expected, matched the glycosylation of unfractionated nMPO from resting neutrophils (PMN-nMPO, r > 0.95, see Figure S5 and Table S6 for all data). Given our recent glycomics-based report of granule-specific *N*-glycosylation of resting neutrophils (38) and the observation of uniform *N*-glycosylation of neutrophil elastase across granules (40), the observation of relatively similar *N*-glycosylation of MPO distributed across granules indicates that both the different granule protein populations and differential glycan processing of the proteins trafficking to the individual granules contribute to the compartment-specific glycosylation of neutrophils.

MPO glycoprofiling of maturing neutrophils, facilitated by data re-interrogation (50), indicated that PM-MPO and MPO from all subsequent neutrophil development steps surprisingly carry fully processed *N*-glycosylation signatures at all sites (r > 0.93). Supporting the MPO maturation in early-stage granulopoiesis, longitudinal profiling of the proMPO-to-MPO conversion within maturing, mature and granule-separated neutrophils showed a near-complete pro- and hexapeptide removal from metamyelocyte-derived MPO (MM-MPO, ∼1% proMPO) and from MPO of more advanced cellular maturation stages, Figure 3*E*. Our data could not discriminate between the apoproMPO and proMPO forms (without and with heme, respectively), but the unprocessed form observed in this study is likely proMPO since heme acquisition reportedly occurs rapidly in the ER (22). Taken together, our data indicate that the heavily processed MPO undergoes rapid glycan and polypeptide maturation upon expression at the PM stage during granulopoiesis.

In addition to the characterisation during neutrophil maturation, we also glycoprofiled MPO during pathogenic infection. *S. aureus*–mediated activation of neutrophils performed at sub-stoichiometric levels to simulate the relatively weak chronic infection levels experienced by individuals with cystic fibrosis and to prevent cell lysis (MOI 1:5) demonstrated a rapid (30–120 min) time-dependent and cell-death-independent degranulation of Dg-MPO, Figure 3*F*. The Dg-MPO carried similar glycosylation to PMN-nMPO (r > 0.91) indicating a glycoform-independent degranulation process of the protein. It remains unknown if the weakly altered glycans observed at selected sites (*e.g.* Asn355, r = 0.81) functionally impact the pathogen-killing ability of MPO or result from technical variation of the analytically challenging site-specific profiling of MPO directly from biological mixtures (52).

Sequence alignments showed that Asn355 and Asn391 are conserved sequons within the family of human peroxidases and within MPO expressed across mammalian species, Figure 3*G* and Figure S6. The sequon conservation of Asn355 and Asn391, which recently was strengthened by the observation of similar glycosylation patterns of these two sites on human and mouse MPO (53), implies a functional relevance of the Asn355- and Asn391-glycans. Interestingly, absence of glycosylation and hyper-truncated GlcNAcβAsn were found to be elevated features of Se/Pl-MPO at Asn355 (*p* = 9.2 × 10^−5^ and 0.04, respectively, Se/Pl- *versus* Az-MPO) and Asn391 (*p* = 0.022 for GlcNAcβAsn) relative to MPO from other granules, Figure 3*H*. It may be speculated that the absence or hyper-truncation of glycans positioned at Asn355 and Asn391 of Se/Pl-MPO arises from the action of endoglycosidase H- (Endo H-) and peptide:*N*-glycosidase F-like hydrolases *e.g.* di-*N*-acetylchitobiase (Q01459) and N(4)-(beta-*N*-acetylglucosaminyl)-L-asparaginase (P20933) and/or other glycoside hydrolases of the truncation pathway that may be co-expressed and co-sorted with MPO trafficking to these compartments (48, 54). Regardless of the underlying biosynthetic processes, the position-specific enrichment of uncommon glycan signatures in specific compartments of the neutrophil is intriguing and without precedence in the literature.

### Hyper-truncated Asn355- and Asn391-GlcNAc residues allosterically augment MPO activity

**Figure 4:**
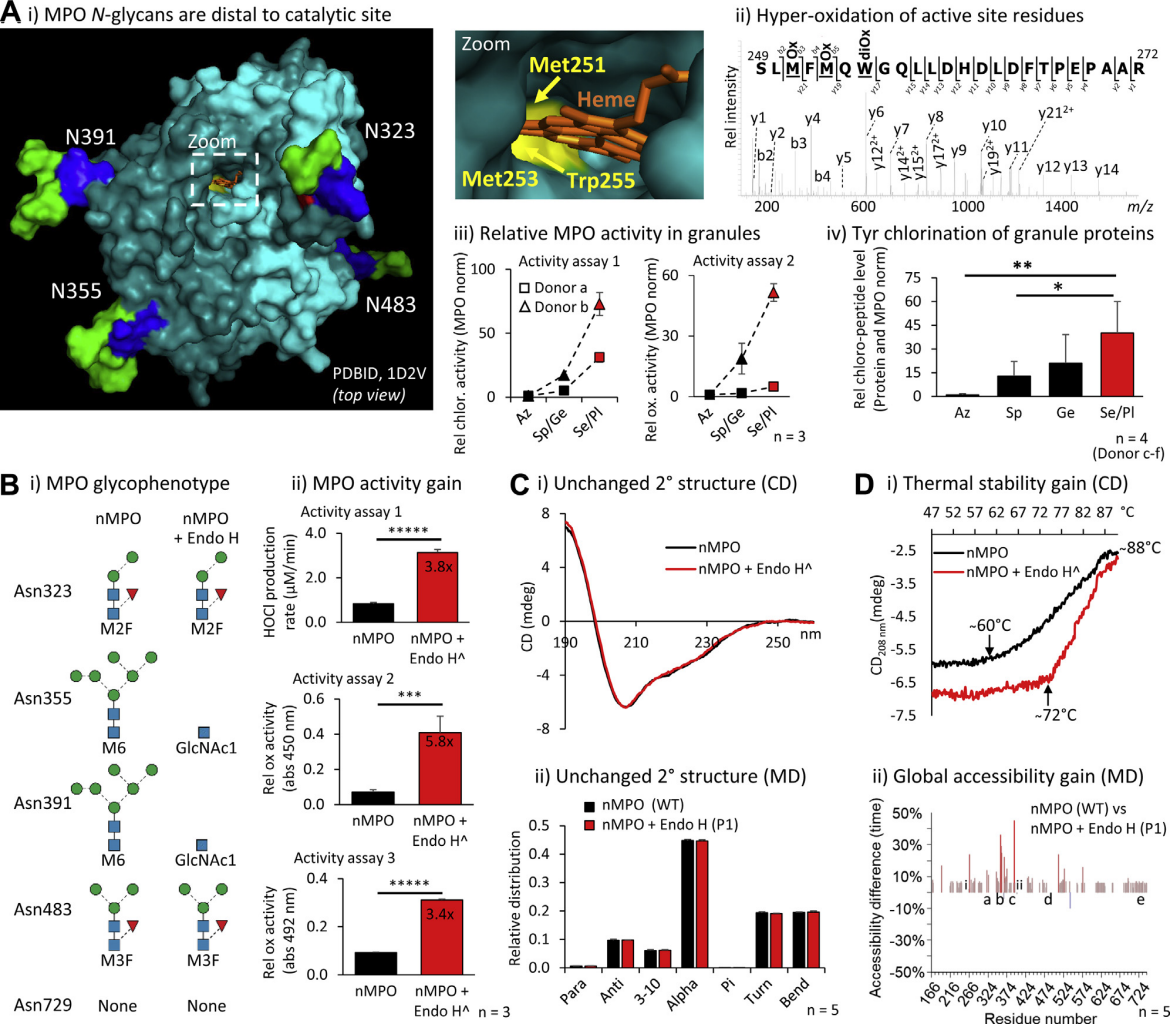
**Hyper-truncated Asn355- and Asn391-GlcNAc signatures positioned distal to the catalytic site augment the MPO activity.***A*, *i*, The MPO *N*-glycans are positioned distal to the catalytic site. Zoom: Oxidation-prone Met251, Met253 and Trp255 (*yellow*) in the heme-containing (*orange*) catalytic site. Mapped on monoprotomeric MPO (PDBID, 1D2V), see Figure 1 for key. *ii*, hyper-oxidation of Met251, Met253 and Trp255 indicates extensive auto-oxidation. *iii*, Se/Pl-MPO from Donor a–b neutrophils displayed a higher chlorination and oxidation activity than MPO from other granules (Table S7). Adjusted for MPO levels, n = 3, technical replicates. *iv*, relative Tyr chlorination level of proteins from granules fractionated with high resolution of neutrophils from Donor c–f (Table S8). Adjusted for total protein and MPO levels, n = 4, biological replicates. *B*, *i*, Endo H-treatment of nMPO produced an Asn355- and Asn391-GlcNAc glycophenotype (mimicking the enriched GlcNAcβAsn signatures of Se/Pl-MPO, see Fig. 3*H*) as validated using LC-MS/MS (Fig. 5*B*). Main glycoforms are depicted for each site. *ii*, the Endo H-treated nMPO exhibited a higher enzyme activity than native nMPO based measurements of the HOCl production (activity assay 1) and the oxidation rate (activity assays 2–3) (all assays, n = 3, technical replicates) (Table S9, *A–C*). *C*, Endo H-treated and untreated nMPO displayed indistinguishable secondary structure profiles based on (*i*) CD profiling (Table S11, *A–B*) and (*ii*) MD-based predictions. *D*, relative to native nMPO (WT), Endo H-treated MPO (P1, see Table S10, *A–B* for overview of the modelled MPO glycoforms) showed (*i*) higher thermal stability as determined by CD_208 nm_ (*arrows* indicate initial melting temperatures) and (*ii*) greater global polypeptide accessibility based on MD data (n = 5, technical replicates) (Figs. S8 and S9). Key: a–e and *i*–*ii* indicate positions of the MPO glycosylation sites and key catalytic residues, respectively. For panel Bii, Ci, and Di: ^∧^Endo H-treated nMPO data were normalised based on Endo H only control data to enable comparison to untreated nMPO. For all panels: Data plotted as mean ± SD, ns, not significant (*p* ≥ 0.05), ∗*p* < 0.05, ∗∗*p* < 0.01, ∗∗∗*p* < 0.005, ∗∗∗∗*p* < 0.001, ∗∗∗∗∗*p* < 0.00005.

Next, we explored the function of MPO glycans, which are unlikely to interfere directly with the substrate–product exchange to the heme-containing catalytic site as these are located distal to the active site, Figure 4*A*i. We therefore investigated the modifications of the catalytic site using peptide-focused LC-MS/MS. Hyper-oxidation of Met251, Met253 and Trp255 lining the catalytic site was observed indicating extensive auto-oxidation that may impact the MPO activity by possibly reconfiguring the active site, Figure 4*A*ii. Activity assays performed on granules of Donor a–b neutrophils fractionated on two-layered density gradients were then used to demonstrate that Se/Pl-MPO exhibits a higher chlorination activity (3.7–9.0-fold) and oxidation activity (5.0–51.5-fold, Se/Pl- *versus* Az-MPO) than MPO from other granules, Figure 4*A*iii and Table S7. In support, peptide analysis of granules from Donor c–f neutrophils fractionated on three-layered density gradients confirmed that Se/Pl-MPO exhibits a higher chlorination activity than MPO from other granules based on a higher Tyr chlorination level of the granule proteins (*p* = 4.7 × 10^−3^, Se/Pl- *versus* Az-MPO), Figure 4*A*iv and Table S8.

Endo H-treatment of nMPO under native conditions produced an Asn355- and Asn391-GlcNAc-rich glycophenotype mimicking the enriched glycoforms of Se/Pl-MPO, Figure 4*B*i. As discussed further below, LC-MS/MS-based characterisation validated that the oligomannose-rich Asn355- and Asn391-glycans were fully converted to GlcNAcβAsn while the processed *N*-glycans in other positions were Endo H-insensitive, Figure S7. Excitingly, the Endo H-treated nMPO exhibited higher chlorination and oxidation activity than untreated nMPO as established using three different activity assays (3.4–5.8×, all *p* < 0.005), Figure 4*B*ii and Table S9, *A–C*.

The molecular basis of the intriguing glycoform-dependent MPO activity was investigated using CD and MD simulations of relevant MPO glycoforms (WT and P1–P3, see Table S10, *A–B* for overview of the modelled MPO glycoforms). Endo H-treated nMPO (P1) and untreated nMPO (WT) displayed indistinguishable secondary structure profiles rich in helical content as reported (55, 56) based on CD data and predictions based on MD data, Figure 4*C* and Table S11, *A–B*. Notably, however, the Endo H-treated nMPO showed a higher initial melting temperature (72 °C) than untreated nMPO (60 °C) as determined by CD_208 nm_, Figure 4*D*i and Table S11*C*. Both glycoforms completed their transition by 88 °C, though about half the helical structure remained at this temperature. Thus, our data suggest that Endo H-treated nMPO displays an enhanced thermal stability relative to native nMPO. MD indicated that the relatively higher thermal stability of Endo H-treated nMPO was accompanied by a significantly higher global polypeptide accessibility relative to nMPO, Figure 4*D*ii and Figures S8 and S9. Similar accessibility gains distributed throughout the polypeptide chains, but with particular “hot-spots” of dramatically enhanced accessibility of residues C-terminal to Asn355 and Asn391 (labelled b–c) and distal to the heme and active site residues (i–ii, His261/Arg405), were observed for P2 (mimicking the Se/Pl-MPO-enriched glycoforms). The P3 glycoform carrying semi-truncated *N*-glycans at Asn355 and Asn391 (M2–M3) did not show elevated accessibility relative to nMPO suggesting that absence or hyper-truncation of glycans positioned at Asn355 and Asn391 is required to allosterically impact the global MPO structure and augment activity. In support, prolonged contacts were consistently observed between the β1,4-GlcNAc and β1,4-Man of the trimannosylchitobiose core (Manβ1,4GlcNAcβ1,4GlcNAcβAsn) and the polypeptide region immediately C-terminal to Asn355 and Asn391 during the MD simulation of glycans elongated beyond the GlcNAcβAsn at these positions (data not shown). Such glycan–protein contacts, which may modulate protein stability and structure, are particularly interesting given that Asn355 and Asn391 are positioned on flexible loops proximal to the active site. The MD data including RMSD-based mobility measurements, however, were insufficiently sensitive to unravel the molecular basis of the observed structure–activity relationships of glycosylated MPO in greater details, which, consequently, await future exploration, Figure S8*D*.

### Hyper-truncated Asn355-glycosylation enhances ceruloplasmin-mediated MPO inhibition

**Figure 5:**
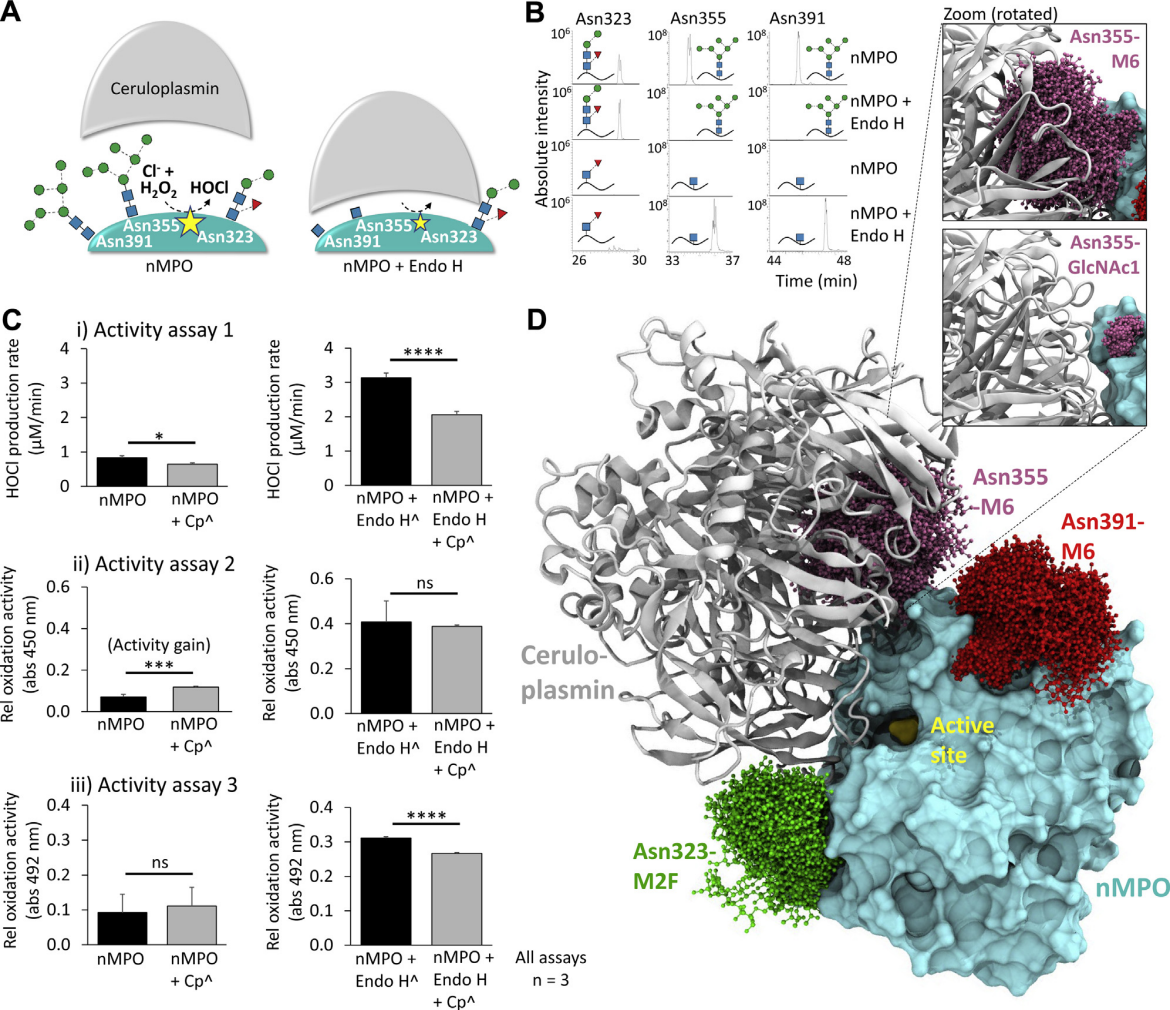
**Hyper-truncated Asn355-glycans augment the ceruloplasmin-mediated MPO inhibition.***A*, schematics of the glycoform-dependent ceruloplasmin-based inhibition of Endo H-treated and untreated nMPO. Our data support that nMPO glycoforms carrying hyper-truncated GlcNAc signatures at Asn355 and Asn391 enable a closer contact to the natural MPO-inhibitor ceruloplasmin thereby sterically precluding an efficient substrate-product exchange to the catalytic site of nMPO as illustrated. Common glycans decorating Asn323, Asn355 and Asn391 in proximity to the MPO-ceruloplasmin interface and the HOCl-producing active site (*yellow star*) are portrayed. *B*, glycopeptide data (selected EICs) demonstrating complete conversion of Asn355-M6 and Asn391-M6 to GlcNAcβAsn upon Endo H-treatment (Fig. S7). The Endo H-insensitive Asn323-M2F was included as a control. *C*, the chlorination (*i*) and relative oxidation (*ii*–*iii*) levels of native nMPO (left graphs) and Endo H-treated nMPO (right) incubated with (*gray bars*) and without (*black*) serum ceruloplasmin (Cp) were determined in technical triplicates using activity assay 1-3, respectively (Table S9, *D–F*). The activity data of nMPO and Endo H-treated nMPO (*black bars*) already shown in Figure 4*B*, *ii* were included again to allow for a comparison to the activity data of the Cp-treated nMPO. ˆEndo H-and Cp-treated nMPO data were normalised based on Endo H and Cp only control data to enable comparison to untreated nMPO (for all assays, n = 3, technical replicates). *D*, MD data of Asn323-M2F (*green*), Asn355-M6 (*magenta*) and Asn391-M6 (*red*) modelled on a crystal structure of the ceruloplasmin–MPO complex (PDBID, 4EJX) demonstrated that Asn355-M6 and not Asn355-GlcNAc clashes with ceruloplasmin (see zoom). Data plotted as mean ± SD, ns, not significant (*p* ≥ 0.05), ∗*p* < 0.05, ∗∗*p* < 0.01, ∗∗∗*p* < 0.005, ∗∗∗∗*p* < 0.001.

The impact of glycosylation on MPO inhibition by ceruloplasmin, an endogenous inhibitor of MPO (27), was explored by comparing the relative activity of Endo H-treated and untreated nMPO in the absence and presence of serum-derived ceruloplasmin, Figure 5*A*. As already discussed above (Fig. 4*B*), the Endo H-treatment completely converted the oligomannose-rich Asn355 and Asn391 to GlcNAcβAsn-containing sites while sites containing processed glycans (*e.g.* Asn323) were Endo H-insensitive, Figures 5*B* and S7. The chlorination and relative oxidation rates of Endo H-treated and untreated nMPO were determined with and without equimolar ceruloplasmin using the three independent activity assays already described above, Figure 5*C* and Table S9, *D–F*. Activity assays 1 and 3 showed significant ceruloplasmin-mediated inhibition of Endo H-treated nMPO (*p* = 3.8 × 10^−4^ and 8.5 × 10^−5^) while untreated nMPO exhibited weak to no ceruloplasmin-mediated inhibition. For activity assay 2, an activity gain was observed for untreated nMPO upon the addition of ceruloplasmin; this unexplained activity gain was quenched for the Endo H-treated nMPO. The molecular mechanisms underpinning the glycoform-dependent MPO inhibition by ceruloplasmin were explored using MD data of relevant Asn323-, Asn355- and Asn391-glycans modelled on a structure of the ceruloplasmin–MPO complex (PDBID, 4EJX), Figure 5*D*. Modelling demonstrated that only Asn355 is positioned directly in the MPO:ceruloplasmin interface, and, importantly, that Asn355-M6 and not Asn355-GlcNAc sterically clashes with ceruloplasmin thereby providing a molecular basis for the glycoform-dependent inhibition of MPO by ceruloplasmin.

## Conclusions

We have characterised the structure–biosynthesis–activity relationship of neutrophil granule MPO and longitudinally profiled the spatiotemporal expression, protein processing, site-specific glycosylation and degranulation of MPO from maturing, mature, granule-separated and pathogen-activated neutrophils from healthy donors. Powered by multi-omics tools, our quantitative and longitudinal data add important molecular-level knowledge to our growing understanding of the complex MPO biology governing many innate immune processes central to human health and disease (41, 57).

Complementary mass spectrometry and novel mass photometry approaches helped unravel the molecular complexity displayed by the heavily glycosylated and post-translationally modified nMPO. Critically, nMPO is site-specifically modified by unique glycans that are rarely reported in human glycobiology, but indeed are common to neutrophils including the hyper-truncated paucimannosidic- and chitobiose core-type *N*-glycans (48, 49), as well as elaborate oxidation, chlorination and polypeptide truncation variants. The structural glycan data presented herein align with and significantly expand on the existing knowledge base (30, 31, 33) by providing not only details of the glycan isomers, but also the molecular mechanisms contributing to the distinctive site-specific MPO glycosylation. Several protein factors including the spatial environment and dimerisation status were found to affect the local *N*-glycan processing producing a position-specific glycan-code, which, intriguingly, was found to impact both the MPO structure and the activity and inhibition potential of the enzyme. The rarely reported GlcNAcβAsn-type glycans were elevated at strategic sites that are important for the activity and ceruloplasmin-mediated inhibition of MPO within mobile compartments of neutrophils.

While glycans are widely recognised to modulate the functions and the physicochemical properties of proteins including, amongst others, the stability, solubility and receptor binding potential of proteins and their susceptibility to proteolysis (58, 59, 60, 61, 62, 63), the literature seemingly harbours relatively few examples of glycans impacting the activity of enzymes such as bovine pancreatic ribonuclease (64) and human tissue plasminogen inhibitor (65, 66). We were unable to find any studies reporting on hyper-truncated *N*-glycans modulating both the enzyme activity and inhibition potential in the literature.

Taken together, our data suggest that neutrophils dynamically produce, process, package, store and, upon activation, release a repertoire of related MPO glycoforms displaying a continuum of different activity and inhibition profiles. The strategically positioned Asn355 glycosylation site carrying both hyper-truncated and elongated *N*-glycans was found to be particularly important for MPO function, a finding that may guide future glycoengineering efforts aiming to generate therapeutically relevant recombinant MPO products with tuneable activity and inhibition potential tailored to specific biomedical applications involving persistent and severe pathogen infections.

In conclusion, this study has provided new molecular-level insights into the intriguingly complex glycobiology of MPO of importance to fundamental neutrophil biology and MPO-mediated immune processes central to human health and disease.

## Experimental procedures

### Donors, neutrophil isolation, granule separation, activation and maturation of neutrophils

#### Donors

Six 40 ml donor pools of buffy coats each obtained from four healthy individuals (six donor pools × four individuals/pool × 10 ml buffy coat/individual) were provided by The Blood Center, Sahlgrenska University Hospital, Gothenburg, Sweden. These six donated buffy coat pools are referred to as ‘Donor a–f’, see Table S12 for an overview of the donor samples, how they were handled and the conducted assays. Further, for the experiments involving pathogen-based activation of neutrophils, three 20 ml non-pooled buffy coats obtained from three healthy individual were provided by The Blood Center, Sahlgrenska University Hospital, Gothenburg, Sweden. These non-pooled buffy coat samples are referred to as “Donor g–i”. Details of the donors of maturing neutrophils used to establish the protein and transcript profiles of immature neutrophils undergoing granulopoiesis based on data reinterrogation (see details below) have been published (50).

#### Neutrophil isolation

Primary neutrophils were isolated from the buffy coat from Donor a-i as previously described (18, 67). Briefly, the erythrocytes were removed by dextran (1%, w/v) sedimentation. Monocytes and lymphocytes were removed by centrifugation at 400*g*, 4 °C, 30 min on a Ficoll–Paque density gradient. The cells were then washed two times for 30 s in Krebs–Ringer buffer with 10 mM aqueous glucose (KRG buffer) to remove the remaining erythrocytes by hypotonic lysis. The neutrophils were then spun at 200*g*, 4 °C, 10 min and resuspended in the KRG buffer. The total neutrophil count for each of the buffy coat pools was ∼10^9^, the purity was >95% and the viability was >99% as measured by a haematology cell counter (Sysmex), trypan blue stain microscopy and Annexin V and propidium iodide staining using flow cytometry (Thermo Fisher Scientific), respectively. Aqueous diisopropyl fluorophosphate was added to a final concentration of 5 mM and kept in the cell suspension for 5 min before excess protease inhibitor was removed by centrifugation at 200*g*, 4 °C, 6 min.

#### Granule separation

Granules were separated from resting neutrophils isolated from Donor a–f. For this purpose, the plasma membranes of the isolated neutrophils were gently disrupted without compromising the integrity of the granule membranes using nitrogen cavitation employing scientific-grade nitrogen (≥99.99% purity, v/v) in a Parr bomb. The released granules from Donor a-b neutrophils were crudely separated using a two-layered Percoll separation method while the released neutrophil granules from Donor c-f were subjected to a three-layered Percoll separation method facilitating high-resolution granule separation as described previously (67, 68). The granule fractionation was validated using granule markers (69), see Extended experimental procedures for details. Granules were lysed and protein extracts collected.

#### Neutrophil activation, MPO degranulation and determination of cell death

Resting neutrophils (Donor g–i) were inoculated with *S. aureus* (LS1, multiplicity-of-infection (MOI) 1:5, bacteria:neutrophils), 37 °C, 0–120 min. Resting neutrophils without *S. aureus* inoculation and with cytochalasin B/ionomycin (CytB/I) and Triton-X 100 stimulation served as activation and a cell death control, respectively. Degranulated MPO (Dg-MPO) and cell death were monitored longitudinally in supernatants using ELISA (ICL LAB) and lactate dehydrogenase (LDH) release using the Cytotoxicity Detection KitPLUS (Sigma), see Extended experimental procedures for details.

#### Maturing neutrophils

Granulopoiesis-related protein and transcript data obtained from maturing neutrophils isolated from four different maturation stages were retrieved from a publicly available data set (50). Immature neutrophils were isolated in their different maturation stages by fluorescence-activated cell sorting from a pool of myeloid progenitor cells derived from the bone marrow of four healthy donors including promyelocytes and myelocytes (collectively referred to as PMs), metamyelocytes (MMs), immature neutrophils with band-formed nuclei (BNs) and mature neutrophils with segmented nuclei (SNs). Circulating (mature) neutrophils (polymorphonuclear cells, PMNs) derived from blood from the same four donors were also investigated, see Extended experimental procedures for details.

#### Neutrophil-derived MPO

Human neutrophil-derived MPO (nMPO, UniProtKB, P05164, >95% purity) was from pooled donor blood (Lee BioSolutions). The purity, concentration, structural integrity and enzyme activity of nMPO were confirmed prior to analysis, see Extended experimental procedures for details.

### Glycan profiling

*N*-glycans were released from nMPO using *Elizabethkingia miricola* peptide-*N*-glycosidase F (Promega) (70). Reduced *N*-glycans were profiled in technical triplicates using porous graphitised carbon (PGC) liquid chromatography–tandem mass spectrometry (LC-MS/MS) in negative ion polarity on an LTQ Velos Pro ion trap mass spectrometer (Thermo Scientific) (71). Glycan fine structures were manually elucidated (72). RawMeat v2.1 (Vast Scientific) and GlycoMod (Expasy) aided the process. *N*-glycans were quantified from area-under-the-curve (AUC) measurements of extracted ion chromatograms (EICs) using Skyline v20.1.0.76 (72), see Extended experimental procedures for details.

### Glycopeptide profiling

Glycopeptides and peptides were profiled from (i) nMPO, (ii) mono- (αβ) and diprotomeric (ααββ)-separated nMPO, (iii) endoglycosidase H- (Endo H-) treated and untreated nMPO, (iv) granule-separated MPO and (v) Dg-MPO released from pathogen-activated neutrophils. For (i) reduced and carbamidomethylated nMPO was digested in technical triplicates using sequencing-grade porcine trypsin (Promega), (ii) mono- and diprotomeric nMPO were separated in technical triplicates using non-reductive SDS-PAGE on a pre-cast 4–12% gradient gel (Invitrogen). Separations were performed using a constant potential of 120 V, 45 min and were carried out on ice to prevent spontaneous dissociation of diprotomeric nMPO. Protein bands were in-gel trypsin digested, (iii) Endo H-treated and untreated nMPO (see below) were applied separately to SDS-PAGE. The β-chains (53–58 kDa) were in-gel trypsin digested, (iv) isolated granule fractions were briefly introduced into SDS-PAGE gels. Bands containing all granule proteins were in-gel trypsin digested, and (v) released proteins were acetone precipitated, reduced, alkylated and in-solution trypsin digested. All peptide mixtures were desalted before LC-MS/MS.

Peptides were separated using C18 chromatography and detected using a Q-Exactive HF-X Hybrid Quadrupole-Orbitrap mass spectrometer (Thermo Scientific) in positive ion polarity. LC-MS/MS data were searched against the canonical human MPO (P05164) and/or the human proteome (all reviewed UniProtKB entries) using Byonic v3.6.0 (Protein Metrics) and MaxQuant v1.6, see Table S13 for overview. Variable modifications including Met and Trp mono-/di-oxidation, Tyr mono-/di-chlorination and *N*-glycan libraries were included in the searches. Glycopeptides with Byonic PEP-2D scores <0.001 were considered and manually validated (73). Non-glycan modified peptides were filtered to peptide-to-spectral matches and protein false discovery rates <0.01 to 0.03 (MaxQuant) or PEP-2D scores <0.001 (Byonic). Glycopeptides were profiled based on AUCs of monoisotopic EICs of glycopeptide precursors using Skyline v20.1.0.76 or Xcalibur v2.2 (Thermo Scientific). Non-glycosylated peptides and proteins were quantified based on precursor intensities using MaxQuant (74), see Extended experimental procedures for details.

### Intact nMPO analysis using native MS and mass photometry

Top-down/native MS was performed of (i) the intact α-chain of nMPO and (ii) intact nMPO. For (i) nMPO was reduced, desalted and injected on a C4 LC column connected to an Agilent 6538 quadrupole-time-of-flight mass spectrometer operating in high-resolution positive polarity mode. Mass spectra were deconvoluted using MassHunter vB.06 (Agilent Technologies). Assignments were guided by the LC-MS/MS peptide data, see Table S3*A* and ii) intact nMPO was infused into a modified Q-Exactive (Thermo Scientific) operating in positive ion polarity *via* nano-ESI using custom-made gold-coated capillaries (75). Data were processed with Xcalibur v2.2 (Thermo Scientific), spectra deconvoluted with UniDec (76) and annotated using in-house software.

Intact nMPO was analysed using single-molecule mass photometry as described (44). Coverslips were assembled for sample delivery using silicone CultureWell gaskets (Grace Bio-Labs). Data were acquired, processed and analysed using in-house software (43), see Extended experimental procedures for details.

### Visualisation, modelling, molecular dynamics, solvent accessibility and sequence alignments

Human diprotomeric MPO (PDBID, 1D2V) was used for visualisation and modelling. Signature *N*-glycans were added *in silico* using the Carbohydrate and Glycoprotein builders within GLYCAM-Web (http://glycam.org) to mimic nMPO (WT), Endo H-treated nMPO (P1), the hyper-truncated Asn355-/Asn391-glycophenotype that was found to be elevated in Se/Pl-MPO (P2) and an MPO glycoform with semi-truncated glycans at Asn355 and Asn391 (P3), see Table S10 for overview. The per-residue solvent accessibilities, root mean squared deviation/fluctuation (RMSD/RMSF) and secondary structures were calculated using Cpptraj (AmberTools 18), plotted with Gnuplot 5.2 and visualised using VMD 1.9.3. Snapshots from the molecular dynamics (MD) simulations of WT- and P1-MPO were structurally aligned to the ceruloplasmin–MPO complex (4EJX) *via* the MPO protein backbone.

Five crystal structures of MPO (PDBID, 1D2V, 1CXP, 1DNU, 1DNW, 5FIW) were used to assess the relative solvent accessibilities to the Asn residue of all sequons of monoprotomeric MPO and to the β1,2-GlcNAc of FA1-glycans at Asn323, Asn483 and Asn729 of mono- and diprotomeric MPO using NACCESS (5 Å radii probe) (77).

Sequence alignments of human MPO (P05164) to (i) the human peroxidase family including eosinophil peroxidase (P11678), lactoperoxidase (P22079) and thyroid peroxidase (P07202) and (ii) MPO from key mammalian species including mouse MPO (P11247), macaque MPO (F7BAA9), porcine MPO (K7GRV6) and bovine MPO (A6QPT4, all downloaded July 2020) were performed using T-Coffee (http://tcoffee.crg.cat/apps/tcoffee) and Boxshade (http://www.ch.embnet.org/software/BOX_form), see Extended experimental procedures for details.

### Endoglycosidase H-treatment of nMPO

Intact nMPO was incubated with or without *Streptomyces plicatus* endoglycosidase H (Endo H, Promega) under native conditions, 37 °C, 16 h. All samples including controls containing only Endo H were used immediately for activity and inhibition profiling and structural characterisation, see Extended experimental procedures for details.

### Chlorination and oxidation activity and ceruloplasmin-mediated inhibition of MPO

Three independent enzyme activity assays designated activity assay 1–3 were carried out to establish the chlorination and oxidation activity of the granule-separated MPO in crude protein mixtures and of the isolated Endo H-treated and untreated nMPO.

Specifically, the chlorination activities of various MPO glycoforms and controls were determined by the formation of HOCl captured *via* taurine per time using HOCl standard curves (activity assay 1) (78). Reactions were initiated by sequential addition of taurine and H_2_O_2_ (Sigma), stopped by catalase (Sigma) and measured at 650 nm after addition of 3,3′,5,5′-tetramethylbenzidine (TMB, Sigma). The relative oxidation activities of various MPO glycoforms and controls were determined using a TMB assay (activity assay 2) and an *o*-phenylenediamine assay (activity assay 3). Reactions were initiated by the addition of TMB or *o*-phenylenediamine (Sigma), quenched after incubation with sulphuric acid (Sigma) and the colour intensity measured at 450 nm or 492 nm, respectively. Readings were adjusted based on water and Endo H controls, Table S9.

Ceruloplasmin-mediated inhibition of the MPO enzyme activity was determined using activity assay 1–3. Endo H-treated and untreated nMPO and controls were incubated with and without human serum-derived ceruloplasmin (P00450, Lee BioSolutions) in technical triplicates prior to activity measurements. Readings were adjusted based on water, Endo H and ceruloplasmin controls, see Extended experimental procedures for details of the MPO activity assays and ceruloplasmin inhibition experiments.

### Circular dichroism profiling and temperature stability

Circular dichroism (CD) data of nMPO, Endo H-treated nMPO and controls were collected in technical duplicates using 1 mm-pathlength cuvettes (Starna Scientific) in a Jasco J-1500 spectropolarimeter. CD spectra were recorded using 260–190 nm scans at pre-melting temperatures (20–50 °C). Thermal stability was determined by monitoring the 208 nm signal over a temperature range. Readings were baseline corrected based on water and Endo H controls, see Extended experimental procedures for details.

### Data representation and statistics

Significance was tested using one-/two-tailed paired/unpaired Student's t-tests. Confidence was designated by ∗*p* < 0.05, ∗∗*p* < 0.01, ∗∗∗*p* < 0.005, ∗∗∗∗*p* < 0.001, ∗∗∗∗∗*p* < 0.0005. ns, non-significant test (*p* ≥ 0.05). Biological and technical replicates have been stated for each experiment. Data were plotted as the mean, while error bars represent their standard deviation (SD).

### Ethics statement

Buffy coats from healthy individuals were provided by The Blood Center, Sahlgrenska University Hospital, Gothenburg, Sweden. According to Swedish law on ethical conduct in human research, ethics approval for buffy coats was not needed because they were provided anonymously and could not be traced back to a specific individual.

## Data availability

This study contains Supporting information. Three supporting files have been provided: (1) Supplementary Information containing Extended experimental procedures and Figures S1–S9, (2) Supplementary Tables containing Tables S1–S13 and (3) Supplementary Data containing Data S1–S3 (PDF).

The LC-MS/MS raw data files are available *via* ProteomeXchange with identifier PXD021131. Username: reviewer83828@ebi.ac.uk, Password: nOuGDU6Q.

## Conflict of interest

The authors declare that they have no conflicts of interest with the contents of this article.

## Supporting information

Extended experimental procedures and Figures S1–S9
